# Effects of transcranial direct current stimulation alone and in combination with rehabilitation therapies on gait and balance among individuals with Parkinson’s disease: a systematic review and meta-analysis

**DOI:** 10.1186/s12984-024-01311-2

**Published:** 2024-02-19

**Authors:** Thi Xuan Dieu Nguyen, Phuc Thi Mai, Ya-Ju Chang, Tsung-Hsun Hsieh

**Affiliations:** 1 School of Physical Therapy and Graduate Institute of Rehabilitation Science, College of Medicine, Chang Gung University, Taoyuan, Taiwan; 2 Healthy Aging Research Center, Chang Gung University, Taoyuan, Taiwan; 3 Neuroscience Research Center, Chang Gung Memorial Hospital Linkou, Taoyuan, Taiwan

**Keywords:** Transcranial direct current stimulation, Rehabilitation therapies, Gait, Balance, Parkinson’s disease

## Abstract

**Background:**

Parkinson’s disease (PD) is a neurogenerative disorder implicated in dysfunctions of motor functions, particularly gait and balance. Transcranial direct current stimulation (tDCS) is a noninvasive brain stimulation offered as a potential adjuvant therapy for PD. This systematic review and meta-analysis were conducted to identify whether tDCS alone and combined with additional rehabilitation therapies improve gait and balance among individuals with PD.

**Methods:**

We searched PubMed, Embase, Web of Science, and relevant databases for eligible studies from inception to December 2022. Studies with a comparative design investigating the effects of tDCS on motor functions, including gait and balance among individuals with PD, were included. A meta-analysis was performed for each outcome using a random effects model for subgroup analysis and pooling of overall effect sizes.

**Results:**

A total of 23 studies were included in the meta-analysis. The pooled results revealed that tDCS has moderate overall effects on gait, measured by gait speed (standardized mean deviation [SMD] = 0.238; 95% confidence interval [CI]  − 0.026 to 0.502); stride length (SMD = 0.318; 95% CI − 0.015 to 0.652); cadence (SMD =  − 0.632; 95% CI − 0.932 to − 0.333); freezing of gait questionnaire scores (SMD =  − 0.360; 95% CI − 0.692 to − 0.027); step length (SMD = 0.459; 95% CI − 0.031 to 0.949); walking time (SMD =  − 0.253; 95% CI − 0.758 to 0.252); stride time (SMD =  − 0.785; 95% CI: − 1.680 to 0.111); double support time (SMD = 1.139; 95% CI − 0.244 to 0.523); and balance, measured by timed up and go (TUG) test (SMD =  − 0.294; 95% CI − 0.516 to − 0.073), Berg balance scale (BBS) scores (SMD = 0.406; 95% CI − 0.059 to 0.87), and dynamic gait index (SMD = 0.275; 95% CI − 0.349 to 0.898). For the subgroup analysis, gait and balance demonstrated moderate effect sizes. However, only cadence, stride time, and TUG indicated a significant difference between real and sham tDCS (*P* = 0.027, *P* = 0.002, and *P* = 0.023, respectively), whereas cadence and BBS (*P* < 0.01 and *P* = 0.045, respectively) significantly differed after real tDCS plus other therapies rather than after sham tDCS plus other therapies.

**Conclusions:**

Our results indicated that tDCS is significantly associated with gait and balance improvements among individuals with PD. The findings of this study provide more proof supporting the effectiveness of tDCS, encouraging tDCS to be utilized alone or in combination with other therapies in clinical practice for PD rehabilitation.

**Supplementary Information:**

The online version contains supplementary material available at 10.1186/s12984-024-01311-2.

## Background

Parkinson’s disease (PD) is the second most common neurodegenerative disorder and the fastest growing in terms of prevalence, disability, and death among neurological diseases, according to the Global Burden of Disease Study reported in 2019 [1–3]. The prevalence of PD increases with age and accounts for up to 4% of individuals in the oldest age groups [4]. PD affects nearly 1% of the population above 60 years old [5] and is expected to increase as the older adult population grows. Consequently, healthcare systems and society are heavily burdened by lost productivity and medical costs [6]. PD is primarily caused by the loss of dopaminergic cells in the substantia nigra pars compacta, which results in reduced dopamine input to the striatum and contributes to excess activation of the inhibitory output of the basal ganglia (BG) [7, 8]. Because the BG is connected with the cortex and cerebellum to form a fundamental circuit, the abnormal inhibition from BG might influence the cortex and cerebellum through the anatomically segregated BG pathway [9–11]. Hence, dysfunction between BG, cortex, and cerebellum (BG–Ctx–Cer) is related to the induction of key PD symptoms, including muscular rigidity, tremor, bradykinesia and postural instability. These motor symptoms can lead to gait and balance deficits, which subsequently can increase fall risk, reduce the quality of life, and increase the mortality rate of patients with PD [11, 12].

Although pharmacology is the gold standard in PD treatment, medications based on dopamine replacement can only control PD and have enormous effects on motor symptoms during the early stages. However, gait and balance are significantly impaired during the late stages and do not respond well to medications such as levodopa [13]. Growing evidence highlights that the potential invasive and noninvasive neuromodulation approaches target various areas in the brain, typically the BG–Ctx–Cer system in patients with PD [11, 14–16].

Transcranial direct current stimulation (tDCS) is a noninvasive brain stimulation technique that applies an anodal or cathodal charge of a weak electrical current over the targeted cortex through two or more electrodes. tDCS can excite or inhibit widespread neuronal activity and trigger dopamine releases through motor networks in the BG–Ctx–Cer system and through other motor cortical areas [14, 17, 18].

Numerous studies have shown that tDCS benefits motor functions, including walking, upper limb functions, and functional locomotion in PD [19–25]. Furthermore, tDCS can be utilized as an adjuvant therapy for PD, often being applied either alone or in combination with with other rehabilitation therapies. However, no systematic review or meta-analysis has specifically explored the effects of tDCS on gait and balance, particularly when tDCS is used as a standalone treatment or in combination with other rehabilitative therapies. In the present systematic review and meta-analysis, we elucidated whether tDCS alone and in combination with other rehabilitation therapies improves gait and balance among individuals with PD. Additionally, we addressed whether the effect of tDCS combined with rehabilitation therapies is superior to rehabilitation therapies. Our findings could provide comprehensive evidence of the effects of tDCS on motor functions and could be valuable for guiding future treatments and research in tDCS.

## Methods

The current systematic review and meta-analysis were performed in accordance with the guidelines of The Preferred Reporting Items for Systematic Reviews and Meta-Analyses (PRISMA) (Additional file 1: Table S1. PRISMA Checklist 2020) [26]. The study protocol was registered with the International Prospective Register of Systematic Reviews under the registration number CRD42022329764 on May 7, 2022.

### Search strategy

Two authors (TXDN and PTM) independently searched three different electronic databases, including PubMed, Embase, and Web of Science, for eligible articles from inception until December 2022. The following terms were used for electronic searching: ((“transcranial direct current stimulation” OR “tDCS” OR “transcranial electrical stimulation” OR “tES”)) AND ((“gait” OR “walking” OR "walk” OR “Spatiotemporal” OR “balance” OR “postural control” OR “postural stability” OR “posture”)) AND ((“Parkinson’s disease” OR “Parkinson” OR “PD” OR “Parkinson disease” OR “Parkinsonism” OR “Parkinsonian”)). Moreover, queries for reference lists of relevant systematic reviews were manually conducted to gather additional eligible studies.

### Selection criteria

Two authors (TXDN and PTM) independently screened the titles, abstracts, and full texts to identify eligible studies for inclusion in this systematic review and meta-analysis. Studies were considered to include if they met the following criteria: (1) recruited patients diagnosed with PD according to UK PD Society Brain Bank clinical diagnostic criteria [27] and did not have comorbid neurological diseases; (2) investigated the effects of tDCS alone or in combination with rehabilitative therapies such as gait training, physical training, dance, aerobic exercises, and strength exercises; (3) included a comparator group comprising PD patients who received sham tDCS, standard care, placebo, or other rehabilitative therapies excluding tDCS; (4) measured outcomes of gait (spatiotemporal gait parameters, freezing of gait questionnaire [FOG-Q], FOG provoking test, walking time, 10-min walking test [10MWT], and 6-m walking test [6MWT]), static balance (center of pressure [CoP] velocity), and dynamic balance (timed up and go [TUG] test, Berg balance scale [BBS], balance evaluation systems test [BESTest], MiniBESTest, functional reach test [FRT], dynamic gait index [DGI], and functional gait assessment [FGA]); (5) were a clinical randomized control trial (RCT), quasi RCT, crossover RCT study, or comparative study; and (6) were published in English.

Studies were considered excluded if they: (1) were a preclinical study; (2) had no control group; (3) were conference abstracts, communications, a letter with no empirical data, or commentary; or (4) did not include the full text.

### Risk of bias and quality assessment

The included studies, which were randomized control trials, were evaluated according to 11 metrics on the Physiotherapy Evidence Database (PEDro) scale [28, 29]. One point was given for each satisfying criterion (except for the first item, which required a YES or NO response). The score ranged from 0 to 10, with a score of 9–10 indicating excellent quality, a score of 6–8 indicating good quality, a score of 4–5 indicating fair quality, and a score of < 4 indicating poor quality. Moreover, the 12-item methodological index for nonrandomized studies [30] was used to evaluate the methodology of nonrandomized studies. The maximum score was 24, and each item was scored from 0 to 2. The higher the score was represented the higher the quality of the study. These scales can be applied to assess the internal and external validity of a clinical trial. Additionally, we identified the evidence level of studies according to the “Oxford Centre for Evidence-Based Medicine 2011 Levels of Evidence” [31], which can assist decision-making in clinical scenarios. The score was independently rated by two authors (TXDN and PTM). Any disagreements on the risk of bias or quality assessments were resolved by a third author or the research team.

### Data extraction

Two authors (TXDN and PTM) performed data extraction independently using a predefined format. Any discrepancies that arose during this process were resolved through discussion. The following data elements were extracted from the included studies: (1) study source (authors, publication year), (2) methods (study designs), (3) participant information (number of participants in each group, mean age, Hoehn & Yahr scores, Unified Parkinson Disease Rating Scale motor section (UPDRS III) scores, medication during the intervention, disease duration), (4) interventions (type of intervention of experimental and control groups, electrode montage, intensity, duration, number of sessions), and (5) outcome measures.

The means, standard deviations (SD), and sample size for each outcome measure were coded and organized in a spreadsheet for meta-analysis [32, 33]. If mean and standard deviations were not available in the included studies, data presented in the form of standard errors, confidence intervals, or medians with ranges were converted into mean and SD format using established statistical formulas as recommended in the literature [34]. In the event of missing data, authors were contacted; if authors did not respond, data values presented as graphs were extracted using the GRABIT software (MathWorks, Inc., Natick, Massachusetts, USA).

### Data synthesis

All statistical data analyses were carried out by Comprehensive Meta-Analysis version 2 software (Biostat, Englewood, NJ, USA). The standardized mean difference (SMD) with 95% confidence intervals (CIs) for each included study was calculated using Cohen’s d method based on the mean and SD. Subsequently, the subgroup analysis for interventions was conducted and the overall effect sizes were pooled for each outcome variable by using a random-effect model. An SMD value of 0.20 or less indicated a small effect size, a value around 0.50 indicated a moderate effect size, and a value of 0.80 or greater indicated a large effect size [35].

The heterogeneity among the results of included studies was determined based on values of *Q* and *I*^2^ statistics [36]. A *P* value of ≤ 0.05 from *Q* statistic and an *I*^2^ value greater than 50% was considered an indicator of significant heterogeneity [37]. If a significant heterogeneity was observed between the studies, the researchers eliminated outliers or subgroups to reduce inconsistencies. We also assessed publication bias through visual inspection of funnel plots and statistical tests, including both Egger’s and Begg’s tests [38, 39], when at least ten studies were included in the meta-analysis following the Cochrane Collaboration guideline [40]. The statistical significance was set at the level of 0.05 (*P* ≤ 0.05) for all calculations.

## Results

### Study identification

**Fig. 1:**
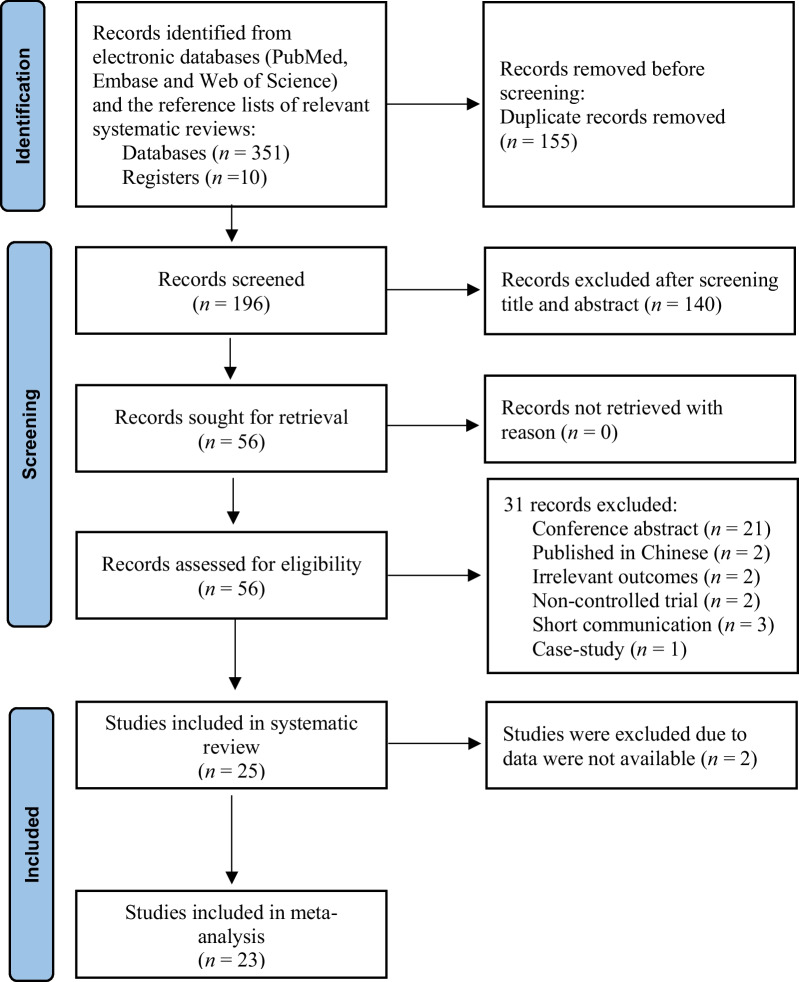
PRISMA flowchart. Literature search and study selection based on inclusion and exclusion criteria from the initiation of search. PRISMA: Preferred Reporting Items for Systematic Reviews and Meta-Analyses

The search yielded a total of 351 records from the PubMed, Embase, and Web of Science databases and the reference lists of relevant systematic reviews (Fig. 1). We then screened titles and abstracts of 196 records after removing 155 duplicates. Altogether, 140 records were excluded. Then, we evaluated the full text of 56 records. After the full-text reading, it is found that 31 texts did not meet the inclusion criteria; 21 records were conference abstracts with no full text available, three were short communications, two were published in Chinese, two produced no relevant outcomes, two were noncontrolled trials, and one was a case study. Overall, 25 studies were eligible and were enlisted in this systematic review. Eleven studies were RCTs, and 14 studies were crossover RCTs. Since two studies were not able to extract appropriate data, a meta-analysis was performed from the data of 23 studies.

### Study characteristics

The demographic characteristics, intervention and comparator descriptions, and outcome measures are illustrated in Table 1.

**Table 1:** The characteristics of included studies in the systematic review

Study		Populations					Intervention (s)		Comparator (s)	Outcome measures
Author	Design	N total (IG/CG)	Mean age	H&Y score	UPDRS III score	Disease duration (yr)	Stimulation protocols	Additional therapies	Stimulation protocols or/and additional therapy	Parameters/scales/tools
Benninger et al., 2010 [45]	RCT	25 (13/12)	63.6 ± 9.0 (IG) 64.2 ± 8.8 (CG)	2.5 ± 0.1 (IG) 2.4 ± 0.2 (CG)	22.2 ± 8.7 (IG) 17.5 ± 8 (CG)	10.6 ± 7.1 (IG) 9.1 ± 3.3 (CG)	atDCS (C3, Fp), 2 mA, 20 min, 8 sessions	None	sham tDCS (forehead), 1 mA, 1–2 min, 8 sessions	Walking time
Beretta et al., 2020 [46]	Crossover RCT	24	68.91 ± 8.47	NR	36 ± 14.32	4.84 ± 3.11	atDCS (C3, C4), 2 mA, 20 min, 1 session	None	Sham tDCS (forehead), 2 mA, 30 s-10 s-30 s, 1 session	Peak of CoP velocity
Bueno et al., 2019 [47]	Crossover RCT	20	64.45 ± 8.9	2.25 ± 0.63	22.35 ± 6.77	7.80 ± 5.32	atDCS (F3), 2 mA, 20 min, 1 session	None	Sham tDCS (F3), 2 mA, 30 s, 1 session	Gait speed, cadence, numb of step, TUG
Criminger et al., 2018 a [42]	Crossover RCT	6	68.13 ± 9.76	NR	23.44 ± 9.73	NR	atDCS (F3), 2 mA, 20 min, 1 session	None	Sham tDCS (F3), 1 mA, 30 s × 2, 1 session	TUG
Dagan et al., 2018a [43]	Crossover RCT	9	68.8 ± 6.8	2.5 ± 0.6	39.7 ± 14.6	9.0 ± 5.7	atDCS (Cz), 1.5 mA, 20 min, 1 session	None	Sham tDCS (Cz, FC1), 0.5 mA, 20 min, 1 session	FOG provoking test, TUG
Dagan et al., 2018b [43]	Crossover RCT	9	68.8 ± 6.8	2.5 ± 0.6	39.7 ± 14.6	9.0 ± 5.7	atDCS (Cz, F3), 1.5 mA, 20 min, 1 session	None	Sham tDCS (Cz, FC1), 0.5 mA, 20 min, 1 session	FOG provoking test, TUG
Kaski et al., 2014a [41]	Crossover RCT	8	NR	NR	NR	NR	atDCS (Cz), 2 mA, 15 min, 1 session	None	Sham tDCS (Cz), 2 mA, 15 min, 1 session	TUG, 6MWT, gait velocity, stride length
Lattari et al., 2017 [48]	Crossover RCT	17	69.18 ± 9.98	2.35 ± 1.06	18.0 ± 8.96	7.06 ± 2.7	atDCS (F3/F4), 2 mA, 20 min, 1 session	None	Sham tDCS (F3/F4), 2 mA, 30 s, 1 session	TUG, BBS, DGI
Manenti et al., 2014 [49]	Crossover RCT	10	67.1 ± 7.2	1.3 ± 1.1	13.3 ± 5.7	8.1 ± 3.5	atDCS (F3/F4), 2 mA, 7 min, 1 session	None	Sham tDCS (F3/F4), 2 mA, 10 s × 2, 1 session	TUG
Manor et al., 2021 [50]	RCT	71 (35/36)	71 ± 8 (IG) 69 ± 7 (CG)	NR	40 ± 14 (IG) 37 ± 17 (CG)	10 ± 6 (IG) 8 ± 6 (CG)	atDCS (F3, Cz), 1.5 mA, 20 min, 10 sessions	None	Sham tDCS, 1.5 mA, 59 s × 2, 10 sessions	TUG, FOG provoking test
Mishra et al., 2021 [51]	Crossover RCT	20	67.8 ± 8.3	1.9 ± 0.9	NR	4.8 ± 3.6	atDCS (F3), 2 mA, 30 min, 1 session	None	Sham tDCS, 2 mA, 30 s, 1 session	Gait speed
Silva et al., 2018 [52]	RCT	21 (11/10)	66 ± 5 (IG) 66 ± 10 (CG)	NR	35.5 (IG) 29.0 (CG) (median)	6 ± 6 (IG) 5 ± 1 (CG)	atDCS (Cz, Fcz), 2 mA, 15 min, 1 session	None	Sham tDCS (Cz, Fcz), 2 mA, 10 s-30 s-10 s, 1 session	Stride length, cadence, gait duration, gait speed
Swank et al., 2016 [53]	Crossover RCT	10	68.7 ± 10.2	2 (median)	24.3	7.9 ± 7.1	atDCS (F3), 2 mA, 20 min, 1 session	None	Sham tDCS (F3), 2 mA, 30 s, 1 session	TUG
Valentino et al., 2014 [54]	Crossover RCT	10	72.3 ± 3.6	2.8 ± 0.5	32 ± 10.3	11 ± 4.9	atDCS (Cz), 2 mA, 20 min, 1 session	None	Sham tDCS (Cz), 2 mA, 30 s × 2, 1 session	Stand walk sit test, FOG-Q, num of FOG, duration of FOG, num of steps
Wong et al., 2022a [44]	RCT	12 (9/3)	54.20 ± 4.1 (IG) 58.30 ± 8.0 (CG)	1.89 ± 0.6 (IG) 1.78 ± 0.7 (CG)	33.22 ± 13.1 (IG) 23.44 ± 14.7 (CG)	93.54 ± 68.2 (IG) 100.18 ± 147.0 (CG) (month)	atDCS (C3), 2 mA, 20 min, 1 session	None	Sham tDCS (C3), 2 mA, 30 s & 60 s, 1 session	Speed, cadence, stride time, stride length, TUG
Wong et al., 2022b [44]	RCT	12 (9/3)	50.09 ± 2.4 (IG) 58.30 ± 8.0 (CG)	1.67 ± 0.5 (IG) 1.78 ± 0.7 (CG)	25.56 ± 17.0 (IG) 23.44 ± 14.7 (CG)	73.81 ± 39.2 (IG) 100.18 ± 147.0 (CG) (month)	atDCS (F3), 2 mA, 20 min, 1 session	None	Sham tDCS (C3), 2 mA, 30 s & 60 s, 1 session	Speed, cadence, stride time, stride length, TUG
Wong et al., 2022c [44]	RCT	12 (9/3)	61.30 ± 7.9 (IG) 58.30 ± 8.0 (CG)	2.13 ± 0.6 (IG) 1.78 ± 0.7 (CG)	24.22 ± 9.9 (IG) 23.44 ± 14.7 (CG)	93.54 ± 68.2 (IG) 100.18 ± 147 (CG) (month)	atDCS (O3/O4), 2 mA, 20 min, 1 session	None	Sham tDCS (C3), 2 mA, 30 s & 60 s, 1 session	Speed, cadence, stride time, stride length, TUG
Chang et al., 2017 [61]	RCT	32 (16/16)	63.6 ± 7.5 (IG) 63.8 ± 8.3 (CG)	NR	NR	4.3 ± 2.5	atDCS (F3), 1 mA, 20 min, 5 sessions	rTMS (simultaneously), 10 Hz, 20 min, 5 sessions	Sham tDCS (F3) + rTMS (10 Hz, 20 min), 5 sessions	FOG-Q, TUG
Conceição et al., 2021 [63]	Crossover RCT	24	70.80 ± 7.87	NR	36.84 ± 14.31	4.3 ± 2.5	atDCS (F3/F4), 2 mA, 20 min, 1 session (after aerobic exercise 10 min)	Aerobic exercise, 30 min, 1 session	Sham tDCS (F3/F4, 2 mA, 30 s, 10 s, 30 s) + aerobic exercise (30 min), 1 session	Swing time variability, step time variability
Costa-Ribeiro et al., 2017 [55]	RCT	22 (11/11)	61.1 ± 9.1 (IG) 62.0 ± 16.7 (CG)	1–4	19.0 (IG) 19.1 (CG)	9.8 ± 4.7 (IG) 9.1 ± 5.3 (CG)	atDCS (Cz), 2 mA, 13 min, 10 sessions (before gait training)	Gait training, 30 min, 10 sessions	Sham tDCS (Cz) + gait training, 43 min, 10 sessions	TUG, BBS, cadence, 10MWT, stride length
Criminger et al., 2018b [42]	Crossover RCT	6	68.13 ± 9.76	NR	23.44 ± 9.73	6.1 ± 3.8 (IG) 6.28 ± 3.74 (CG)	tDCS during bike (F3), 2 mA, 20 min, 1 session	Bike (stationary bicycle), 20 min, 1 session	Sham tDCS (F3, 1 mA, 30 s × 2) + bike (20 min), 1 session	TUG
Criminger et al., 2018c [42]	Crossover RCT	6	68.13 ± 9.76	NR	23.44 ± 9.73	6.1 ± 3.8 (IG) 6.3 ± 3.7 (CG)	atDCS during Wii (F3), 2 mA, 20 min, 1 session	Wii game, 20 min, 1 session	Sham tDCS (F3, 1 mA, 30 s × 2) + Wii game (20 min), 1 session	TUG
Kaski et al., 2014b [41]	Crossover RCT	8	NR	NR	NR	NR	atDCS (Cz), 2 mA, 15 min, 1 session	gait training, 15 min, 1 session	sham tDCS (Cz) + physical training, 15 min, 1 session	TUG, 6MWT, gait velocity, stride length
Lee et al., 2021 [65]	RCT	30 (15/15)	70 ± 3.76 (IG) 1.33 ± 3.27 (CG)	2.47 ± 0.52 (IG) 2.8 ± 0.41 (CG)	34.2 ± 7.82 (IG) 38.67 ± 9.6 (CG)	7	atDCS (Fcz), 2 mA, 20 min, 20 sessions	Visual cueing training, 20 min, 20 sessions	sham tDCS (Fcz) + visual cueing training, 20 min, 20 sessions	FGA, FOG-Q, gait parameters
Manenti et al., 2016 [58]	RCT	20 (10/10)	69 ± 9.1 (IG) 69.1 ± 5.6 (CG)	2.2 ± 0.6 (IG) 2.3 ± 0.4 (CG)	27.8 ± 13.9 (IG) 27.6 ± 8.9 (CG)	NR	atDCS (F3/F4), 2 mA, 25 min, 10 sessions (during physical therapy)	Physical therapy, 25 min, 10 sessions	Sham tDCS (F3/F4) + physical therapy, 25 min, 10 sessions	TUG, four square step test, standing stork test, sit and reach test
Mishra et al., 2022 [64]	Crossover RCT	20	67.8 ± 8.3	1.9 ± 0.9	NR	4.8 ± 3.8	atDCS (F3), 2 mA, 30 min, 1 session (during TUG single and dual task)	TUG single and dual task, 30 min, 1 session	sham tDCS (F3) + TUG single and dual task	TUG
Na et al., 2022 [56]	RCT	23 (11/12)	63.73 ± 6.57(IG) 65.08 ± 6.46 (CG)	1 (IG) 2 (CG) **(**median)	33.64 ± 16.06 (IG) 34.5 ± 12.67 (CG)	6.27 ± 1.03 (IG) 7 ± 1.41 (CG)	atDCS (Cz), 2 mA, 20 min, 10 sessions (first 20 min of 30 min training)	Treadmill gait training, 30 min, 10 sessions	Sham tDCS (Cz) + treadmill gait training, 30 min, 10 sessions	TUB, BBS, FOG, 10MWT, DGI, FRT
Fernández-Lago et al., 2017 [57]	Crossover RCT	18	73.28	1.65	21.17	NR	atDCS (C3/C4), 2 mA, 20 min, 1 session (during treadmill walking)	Treadmill training, 20 min, 1 session	Sham tDCS (C3/C4) + treadmill training, 20 min, 1 session	Gait speed, stride length,
Papen et al., 2014 [62]	Crossover RCT	10	64 ± 10	NR	29 ± 2	6.17	atDCS (C3/C4), 1 mA, 10 min, 1 session	rTMS, 1 Hz, 15 min, 1 session (immediately after tDCS)	Sham tDCS (C3/C4, 1 mA, 5 s) + rTMS (1 Hz, 15 min), 1 session	Number of steps, step length, double support, stride length, cadence
Schabrun et al., 2016 [59]	RCT	16 (8/8)	72 ± 4.9 (IG) 63 ± 11.0 (CG)	2 (median)	47.7 ± 7.5 (IG) 37.7 ± 9.8 (CG)	6.17	atDCS (C3), 2 mA, 20 min, 9 sessions (first 20 min of 60 min training)	Physical therapy, 60 min, 9 sessions	Sham tDCS (C3) + physical therapy, 60 min, 9 sessions	Gait velocity, TUG
Yotnuengnit et al., 2018 [60]	RCT	40 (20/20)	68.2 ± 9.8 (IG) 62.7 ± 8.8 (CG)	2.5 (median)	11.94 ± 4.68 (IG) 11.17 ± 3.97 (CG)	NR	atDCS (Cz), 2 mA, 30 min, 6 sessions (during physical therapy)	Physical therapy, 30 min, 6 sessions	Sham tDCS (Cz) + physical therapy, 30 min, 6 sessions	Gait speed, step length, step width, cadence

All participants were in ON medication state during experiments

10MWT: ten-meter walking test; 6MWT: six minutes waking test; atDCS: anodal tDCS was applied over the target cortex area; BBS: Berg balance scale; CoP: center of pressure; DGI: dynamic gait index; FGA: functional gait assessment; FOG provoking test: freezing of gait provoking test; FOG-Q: freezing of gait questionnaire; FRT: functional reach test; H&Y score: Hoehn and Yahn score; IG/CG: intervention group/control group; NR: not reported; RCT: randomized control trial; TUG: timed up and go test; UPDRS III: Unified Parkinson Disease Rating Scale Motor section

#### Participants

In total, 569 individuals with PD across the included studies were included, with an average age of 50 and 79 years. Of the total number of participants included, the mean Hoehn & Yahr (H&Y) scores were from 1 to 4, the mean PD duration extended from 1.2 to 17.7 years, and the UPDRS III scores ranged from 7.2 to 55.2. All participants were in an ON-medication state for the entire study.

#### Interventions

Among 25 studies included in the systematic review, four of which [41–44] included more than one comparison. Seventeen trials used real tDCS compared with sham tDCS [41–54], and another fourteen trials compared real tDCS plus other rehabilitative therapies with sham tDCS plus other rehabilitation therapies, such as gait training [41, 55–57], physical therapy [58–60], repetitive transcranial magnetic stimulation (rTMS) [61, 62], aerobic exercise [63], dual-task [51, 64], visual cueing [65], and biking and Wii games [42]. In the studies that combined two interventions, the participants received tDCS protocols either simultaneously with or before with other therapies. Anodal tDCS electrodes were mainly placed over different target areas of the motor cortex (the primary motor cortex, dorsolateral prefrontal cortex, or supplementary motor cortex) according to the 10–20 international electroencephalography system. Most studies offered single-session interventions, and the frequency of intervention in other nine studies extended from 5 to 20 sessions [45, 50, 55, 56, 58–61, 65]. The total intervention duration per session ranged between 7 and 60 min, in which the most minor and most prolonged periods of tDCS were 7 [49], and 30 min [51, 60], respectively.

#### Outcomes

Among the gait spatiotemporal parameters, gait speed was included the most (13 studies), followed by cadence (10 studies), stride length (10 studies), and other parameters (step length, walking time, step time, and double support time). Additionally, the FOG-Q was used in four studies, and the FOG provoking test was used in two studies to measure the FOG severity score. Test duration was also used to assess FOG status during walking. However, only one study evaluated static balance by using peak CoP velocity. TUG tests were conducted in 14 studies, and BBSs were used in three studies to measure dynamic balance. Finally, two studies used the DGI to measure balance.

### Risk of bias and quality of included studies

Since all included studies were RCTs, PEDro scale was used to evaluate the risk of bias in each included study. The average score was 7.08 ± 1.11, indicating good quality. In total, three studies scored a 9, indicating excellent quality; 20 studies scored 6–8, indicating that 80% of studies demonstrated good quality; and two studies demonstrated fair quality (Table 2). Only five studies reported allocation concealment [47, 50, 55, 59, 61], and two studies (8%) [50, 59] used an intention-to-treat analysis. Assessors and participants could not be blinded in 10 and 3 studies, respectively. Although blinded therapists often face challenges during the intervention, eight studies (32%) reported success in including blinded therapists. All 25 studies were determined to be level 2 on the "Oxford Centre for Evidence-Based Medicine Levels of Evidence".

**Table 2:** Methodological quality of included studies based on Physiotherapy Evidence Database (PEDro) scale

Study	Point estimates & variability	Between group comparison	Intention to treat	Adequate follow-up	Blind assessors	Blind therapists	Blind subjects	Baseline comparability	Concealed allocation	Random allocation	Eligibility criteria*	Total score	Methodological quality
Benninger et al., 2010 [45]	1	1	0	1	1	0	1	1	0	1	Y	7	Good
Beretta et al., 2020 [46]	1	1	0	1	0	1	1	1	0	1	Y	7	Good
Bueno et al., 2019 [47]	1	1	0	1	0	1	1	1	1	1	Y	8	Good
Chang et al., 2017 [61]	1	1	0	1	1	0	1	1	1	1	Y	8	Good
Conceição et al., 2021 [63]	1	1	0	1	0	0	1	1	0	1	Y	6	Good
Costa-Ribeiro et al., 2017 [55]	1	1	0	1	1	1	1	1	1	1	Y	9	Excellent
Criminger et al., 2018 [42]	1	1	0	1	0	1	0	1	0	1	Y	6	Good
Dagan et al., 2018 [43]	1	1	0	1	1	1	1	1	0	1	Y	8	Good
Fernández-Lago et al., 2017 [57]	1	1	0	1	0	0	0	1	0	1	Y	5	Fair
Kaski et al., 2014 [41]	1	1	0	1	1	0	1	1	0	1	Y	7	Good
Lattari et al., 2017 [48]	1	1	0	1	1	0	1	1	0	1	Y	7	Good
Lee et al., 2021 [20]	1	1	0	1	0	0	1	1	0	1	Y	6	Good
Manenti et al., 2014 [49]	1	1	0	1	1	0	1	1	0	1	Y	7	Good
Manenti et al., 2016 [58]	1	1	0	1	1	1	1	1	0	1	Y	8	Good
Manor et al., 2021 [50]	1	1	1	1	1	0	1	1	1	1	Y	9	Excellent
Mishra et al., 2021 [51]	1	1	0	1	1	1	1	1	0	1	Y	8	Good
Mishra et al., 2022 [64]	1	1	0	1	0	1	1	1	0	1	Y	7	Good
Na et al., 2022 [56]	1	1	0	1	1	0	1	1	0	1	Y	7	Good
Papen et al., 2014 [62]	1	1	0	1	0	0	0	1	0	1	Y	5	Fair
Schabrun et al., 2016 [59]	1	1	1	1	1	0	1	1	1	1	Y	9	Excellent
Silva et al., 2018 [16]	1	1	0	1	1	0	1	1	0	1	Y	7	Good
Swank et al., 2016 [53]	1	1	0	1	0	0	1	1	0	1	Y	6	Good
Valentino et al., 2014 [54]	1	1	0	1	0	0	1	1	0	1	Y	6	Good
Wong et al., 2022 [44]	1	1	0	1	1	0	1	1	0	1	Y	7	Good
Yotnuengnit et al., 2018 [60]	1	1	0	1	1	0	1	1	0	1	Y	7	Good

Yes (Y) = 1 point, No (N) = 0 point; * = not included in total score; < 4 = Poor, 4–5 = Fair, 6–8 = Good, 9–10 = Excellent

### Effects of tDCS alone and in combination with rehabilitation therapies

#### Results of subgroup analysis

The effects of tDCS for each outcome are summarized in Table 3.

**Table 3:** The results of subgroup analysis

Outcome measures	Subgroup	*N*	SMD	Lower limit	Upper limit	*P*-value
Gait						
Gait speed	Alone tDCS	7	0.249	− 0.164	0.660	0.449
	Combined tDCS	6	0.231	− 0.112	0.574	0.187
Stride length	Alone tDCS	5	0.325	− 0.223	0.873	0.246
	Combined tDCS	5	0.315	− 0.106	0.736	0.143
Cadence	Alone tDCS	5	− 0.570	− 1.075	− 0.066	0.027*
	Combined tDCS	5	− 0.666	− 1.039	− 0.294	< 0.001**
FOG− Q	Alone tDCS	2	− 0.375	− 0.815	0.064	0.094
	Combined tDCS	2	− 0.338	− 0.847	0.17	0.192
Step length	Alone tDCS	–	–	–	–	–
	Combined tDCS	3	0.459	− 0.031	0.949	0.066
Walking time	Alone tDCS	3	− 0.253	− 0.758	0.252	0.327
	Combined tDCS	–	–	–	–	–
Stride time	Alone tDCS	3	− 1.262	− 2.073	− 0.450	0.002**
	Combined tDCS	1	− 0.347	− 1.068	0.374	0.345
Double support time	Alone tDCS	–	–	–	–	–
	Combined tDCS	3	1.139	− 0.244	2.523	0.107
Balance						
TUG	Alone tDCS	12	− 0.335	− 0.624	− 0.045	0.023*
	Combined tDCS	8	− 0.237	− 0.582	0.108	0.178
BBS	Alone tDCS	1	0.144	− 0.529	0.817	0.675
	Combined tDCS	2	0.621	0.014	1.227	0.045*
DGI	Alone tDCS	1	0.292	− 0.665	1.249	0.550
	Combined tDCS	1	0.262	− 0.349	1.084	0.552

Alone tDCS: real tDCS versus sham tDCS; Combined tDCS: real tDCS plus other therapies versus sham tDCS with other therapies; BBS: Berg balance scale; DGI: dynamic gait index; FOG− Q: freezing of gait questionnaire; SMD: standardized mean deviation; tDCS: transcranial direct current stimulation; TUG: timed up and go test. *: *P* < 0.05; **: *P* < 0.01

#### Real tDCS versus sham tDCS

The effects of tDCS alone on gait were assessed by measuring gait speed (seven studies), stride length (five studies), cadence (five studies), FOG-Q (two studies), walking time (three studies), and stride time (three studies). Compared with a control group receiving sham tDCS, PD patients in the real tDCS group exhibited greater gait speed and stride length and lower cadence, FOG-Q, walking time, and stride time with moderate effect sizes. Real tDCS significantly affected the decrease in cadence and stride time (*P* = 0.027 and *P* = 0.002, respectively). To evaluate the effect of tDCS alone on balance, 12 studies used TUG tests, one used the BBS, and one used the DGI. The results indicated that real tDCS is associated with greater balance. However, a statistically significant difference was found only in the TUG tests (*P* = 0.023).

#### Real tDCS plus other therapies versus sham tDCS with other therapies

The effects of tDCS with other therapies on gait were assessed by measuring gait speed (six studies), stride length (five studies), cadence (five studies), FOG-Q (two studies), step length (three studies), stride time (one studies), and double support time (three studies). The effects on balance were assessed using TUG tests (eight studies), BBS scores (two studies), and the DGI (one study). The pooled results indicated that the participants in the tDCS plus other therapies group exhibited greater improvements in gait (cadence, *P* < 0.01) and balance (BBS, *P* = 0.045) than those in the sham tDCS with other therapies group, indicating that tDCS can induce additional effects and promote other therapies in PD rehabilitation.

### Overall effects of tDCS

#### Gait

**Fig. 2:**
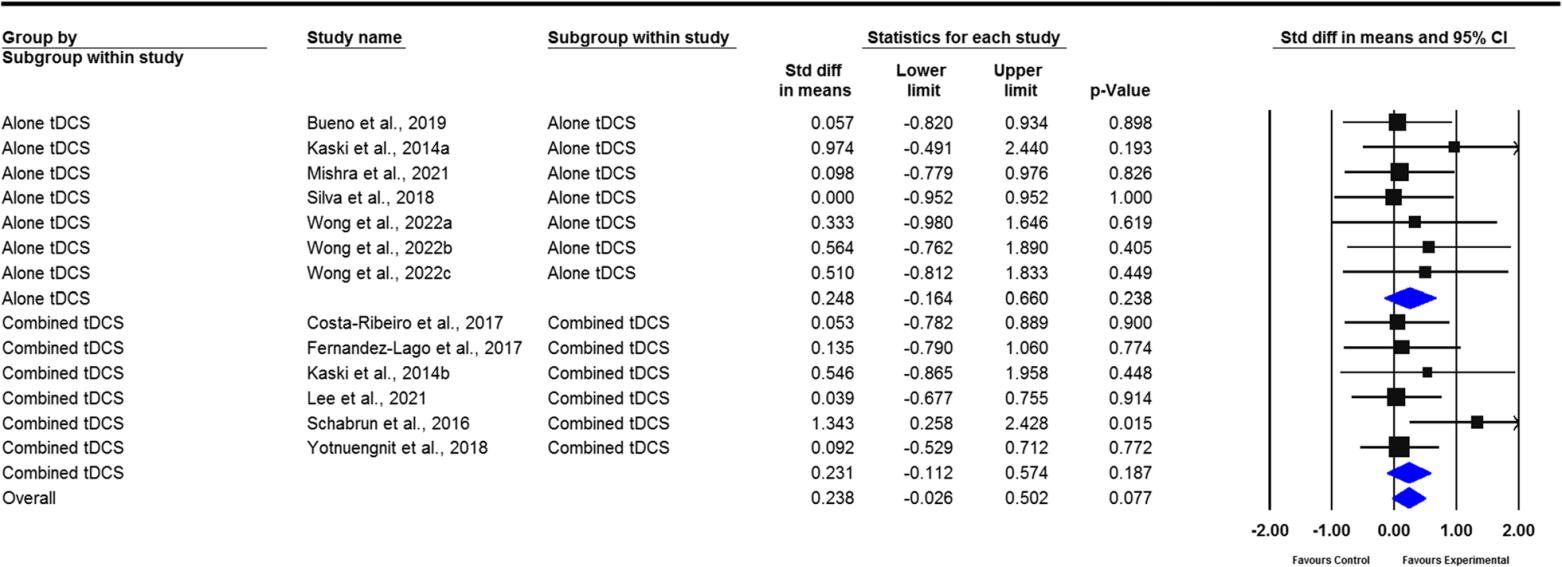
Forest plot of standardized mean difference (SMD) and their 95% CI for gait speed. Black squares represent the SMD in individual trials. Horizontal lines represent 95% confidence interval (CI). The blue rhombus at the bottom indicates an overall pooled effect. tDCS: Transcranial direct current stimulation. The subjects received real tDCS showing an improvement in gait speed. However, this improvement did not reveal statistical significance compared to sham treatment patients (*P* = 0.077)

**Fig. 3:**
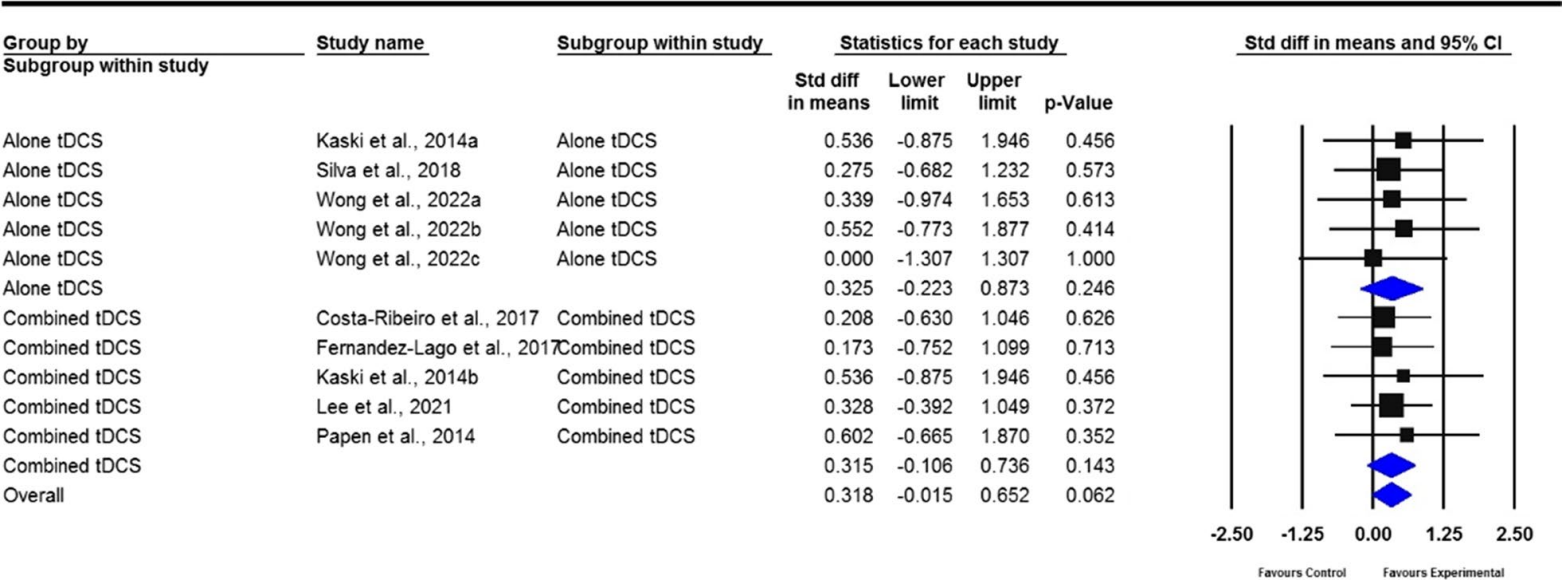
Forest plot of standardized mean difference (SMD) and their 95% CI for stride length. Black squares represent the SMD in individual trials. Horizontal lines represent 95% confidence interval (CI). The blue rhombus at the bottom indicates an overall pooled effect. tDCS: Transcranial direct current stimulation. Similarly, the subjects in real tDCS showed an improvement in stride length. However, this improvement did not reveal statistical significance compared to patients in the sham treatment group (*P* = 0.062)

**Fig. 4:**
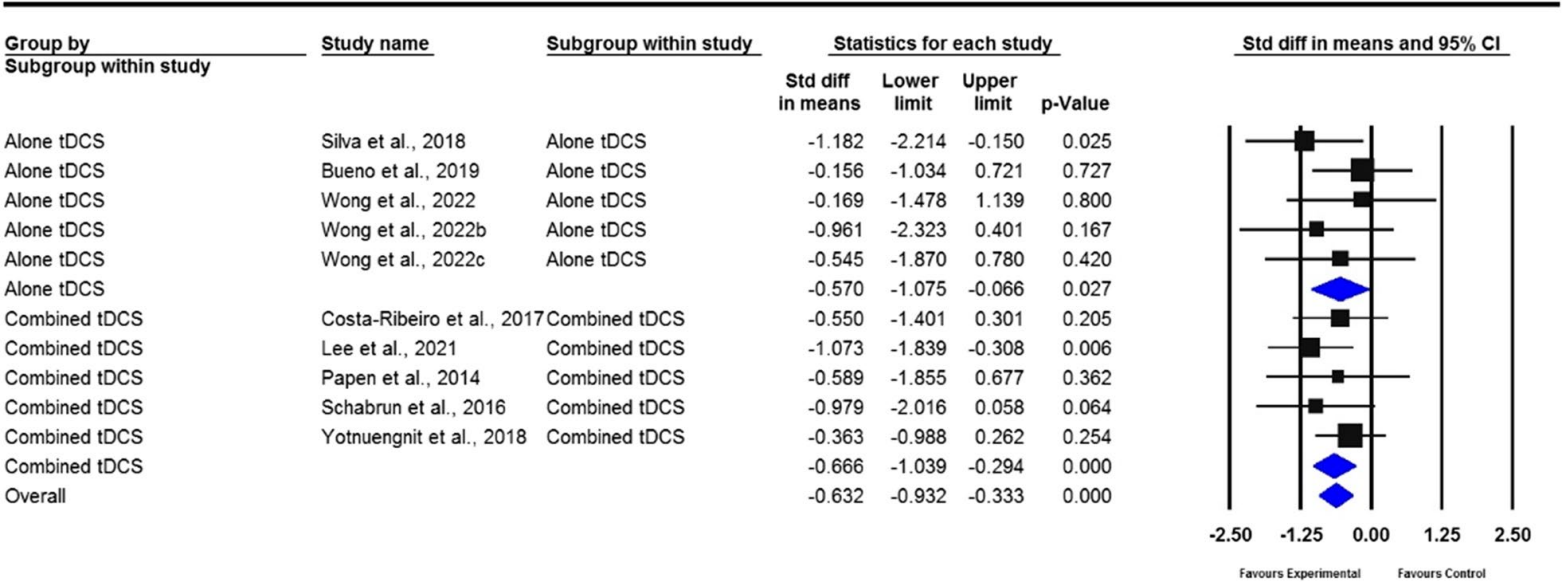
Forest plot of standardized mean difference (SMD) and their 95% CI for cadence. Black squares represent the SMD in individual trials. Horizontal lines represent 95% confidence interval (CI). The blue rhombus at the bottom indicates an overall pooled effect. tDCS: Transcranial direct current stimulation. Subjects who received either real tDCS alone or combined with additional therapies had distinctly reduced cadence during walking. This shows strong evidence that tDCS has a substantial beneficial effect on cadence parameters (*P* < 0.001)

**Fig. 5:**
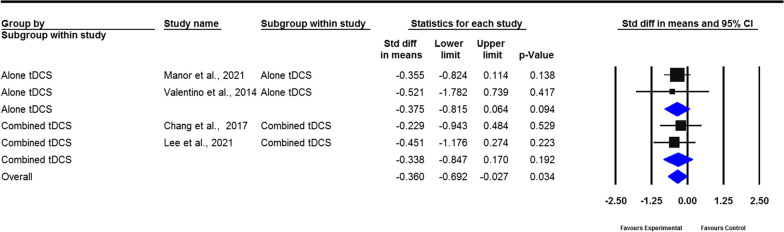
Forest plot of standardized mean difference (SMD) and their 95% CI for freezing of gait questionnaire. Black squares represent the SMD in individual trials. Horizontal lines represent 95% confidence interval (CI). The blue rhombus at the bottom indicates an overall pooled effect. tDCS: Transcranial direct current stimulation. The pooled results showed that tDCS indeed reduces the freezing during gait as measured by the freezing of gait questionnaire with a moderate effect size of 0.360 (*P* = 0.034)

The results of the pooled analysis revealed the moderate effects of the tDCS group on the changes in gait speed (SMD = 0.238; 95% CI − 0.026 to 0.502), stride length (SMD = 0.318; 95% CI − 0.015 to 0.652), cadence (SMD =  − 0.632; 95% CI − 0.932 to − 0.333), FOG-Q (SMD =  − 0.360; 95% CI − 0.692 to − 0.027), step length (SMD = 0.459; 95% CI − 0.031 to 0.949), walking time (SMD =  − 0.253; 95% CI − 0.758 to 0.252), stride time (SMD =  − 0.785; 95% CI − 1.680 to 0.111), and double support time (SMD = 1.139; 95% CI − 0.244 to 0.523). However, only cadence and FOG-Q significantly improved after tDCS compared with the control group (*P* < 0.001, *P* = 0.034, respectively) (Figs. 2, 3, 4, 5, Additional file 2: Figs. S1–4). No heterogeneity was present among studies for all outcome measures of gait (*I*^2^ = 0%, *P* > 0.05). Publication bias was assessed through funnel plot, Egger’s, and Begg’s tests. The analyses revealed that Egger’s test (*P* = 0.018) and Begg’s test (*P* < 0.001) indicated a significant publication bias for gait speed, with one study falling outside the funnel plot. This outlier study included a lengthier intervention protocol than the other studies, which involved three weeks of tDCS combined with dual-task gait training. In addition, no publication bias was observed for cadence and stride length (Additional file 3: Figs. S5–7).

#### Balance

**Fig. 6:**
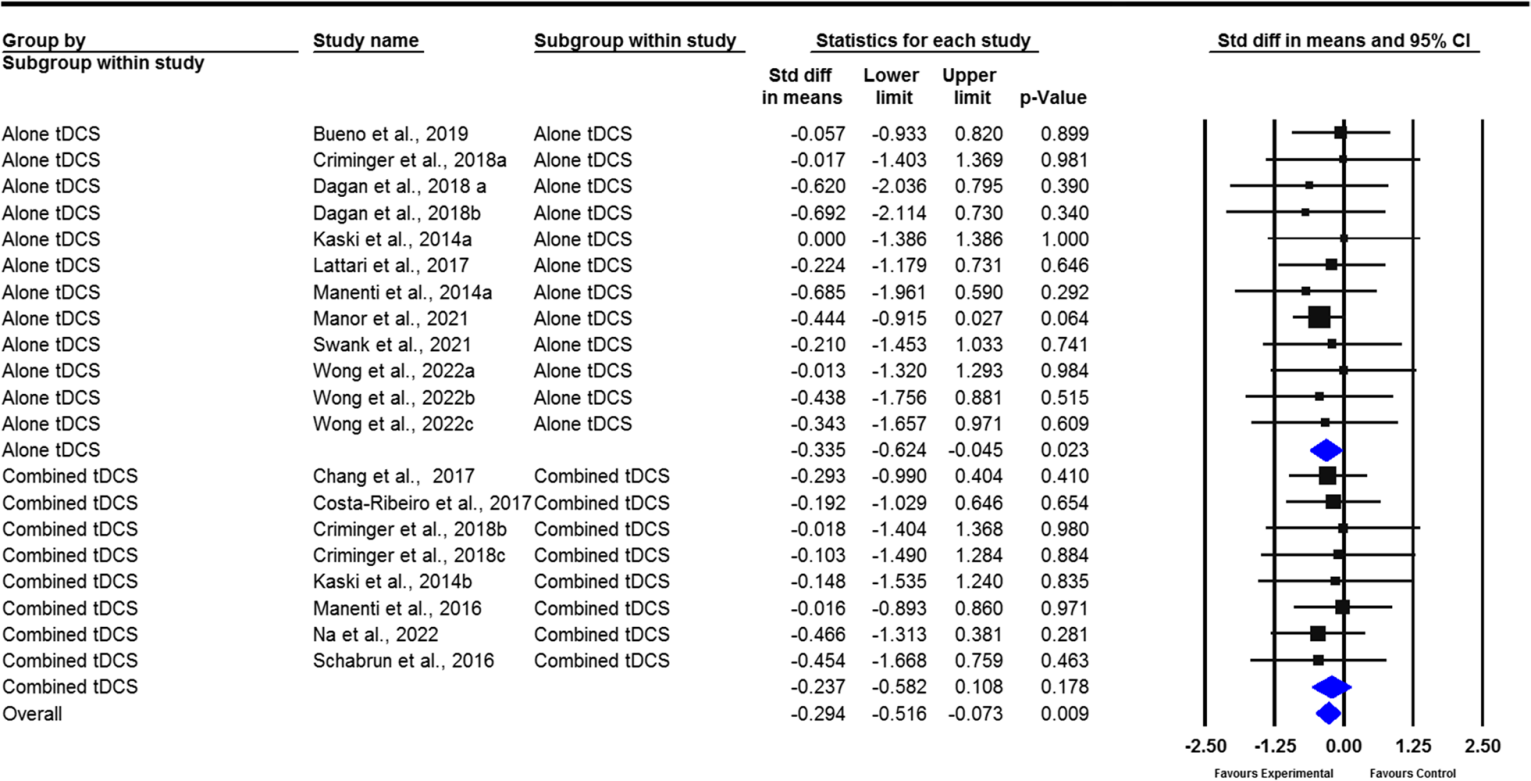
Forest plot of standardized mean difference (SMD) and their 95% CI for timed up and go test. Black squares represent the SMD in individual trials. Horizontal lines represent 95% confidence interval (CI). The blue rhombus at the bottom indicates an overall pooled effect. tDCS: Transcranial direct current stimulation. The results of this meta-analysis show robust evidence that tDCS significantly improved the balance of PD patients compared with controls, as indicated by a reduction in the time required to complete the TUG test (*P* = 0.009)

**Fig. 7:**
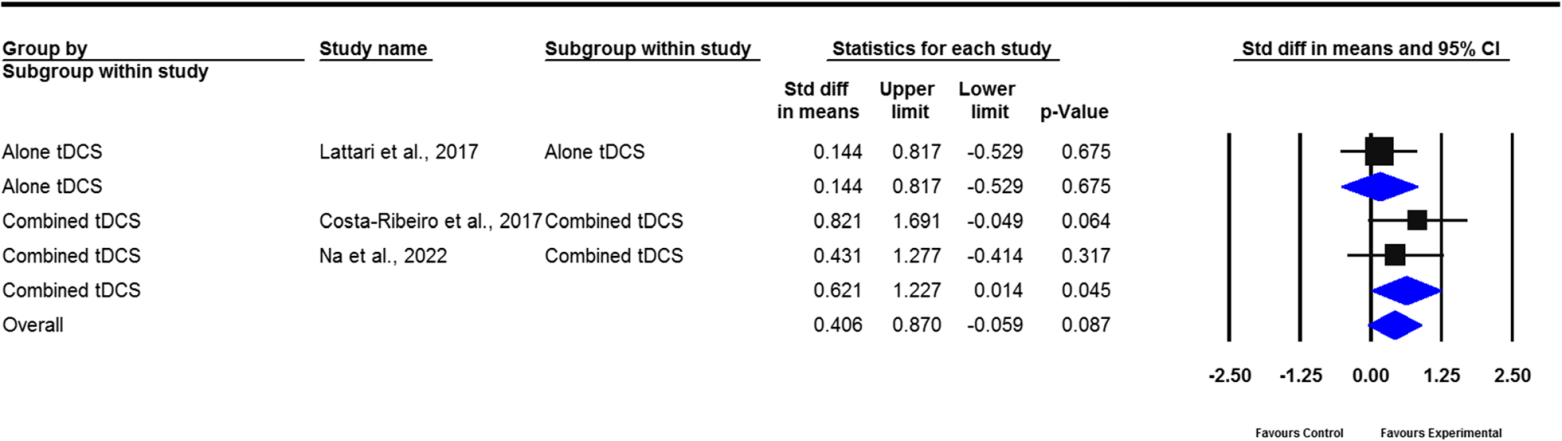
Forest plot of standardized mean difference (SMD) and their 95% CI for Berg balance scale. Black squares represent the SMD in individual trials. Horizontal lines represent 95% confidence interval (CI). The blue rhombus at the bottom indicates an overall pooled effect. tDCS: Transcranial direct current stimulation. The overall meta-analysis result from studies which compared with patients in the sham group with patients who received either tDCS alone or tDCS combined with additional rehabilitation therapies did not show a significant improvement in balance measured by Berg balance scale (*P* = 0.087)

tDCS remarkably improved the balance of PD patients compared with the control group, which was indicated by the decrease in time required to complete the TUG test (SMD =  − 0.294; 95% CI − 0.516 to − 0.073, *P* = 0.009, Fig. 6). Additionally, the meta-analysis results revealed a nonsignificant difference in BBS scores (SMD = 0.406; 95% CI − 0.059 to 0.87, *P* = 0.087, Fig. 7) and the DGI (SMD = 0.275; 95% CI − 0.349 to 0.898, *P* = 0.388, Additional file 4: Fig. S8) between the tDCS group and control group. No publication bias (*P* > 0.05 in Egger’s and Begg’s tests) for the timed up and go test (Additional file 5: Fig. S9) or no heterogeneity (*I*^2^ = 0%, *P* > 0.05) was present among the included studies for all outcome measures.

## Discussion

The current systematic review and meta-analysis summarized the available data on the effectiveness of tDCS alone and in combination with other therapies for patients with PD. Although two studies provided figures with data, we were unable to extract data by using GRABIT; consequently, the data were not included in the meta-analysis [46, 64]. Therefore, we conducted a meta-analysis on 11 outcome measures, including 75 comparisons from 23 studies. Studies were scored from fair quality to excellent quality. Evidence supported that tDCS-induced therapeutic effects play a critical role in managing the motor symptoms of patients with PD. Altogether, the key findings of this review indicated that tDCS protocols greatly affect the gait and balance of patients with PD who are over 50 years old and with mild to severe disease for less than 17 years.

To our knowledge, six meta-analyses [19, 20, 24, 66–68] have been conducted on the effects of tDCS on motor function among patients with PD. These meta-analyses focused on specific aspects of tDCS, such as single versus multitarget regions [24] and real *versus* sham tDCS combined with gait training [19, 68]. In a meta-analysis of 21 studies that enrolled 736 patients with PD, the authors reported insufficient evidence to conclude that tDCS could improve motor functions [67]. The authors proposed that several factors contributed to the tDCS-induced effects on motor functions, including tDCS parameters, stimulation areas, and patient features. Nevertheless, our findings are in agreement with those of other studies [20, 66] that revealed the therapeutic effects of tDCS on gait, balance, and functional mobility but did not reveal any significant difference compared with the control group. However, our meta-analysis was more rigorous than other meta-analyses. We included the broadest range of studies and outcome measures to provide comprehensive evidence that can support decision-making in clinical practices. Additionally, the subgroup analyses were performed to examine the effects of tDCS with and without other therapies, which can benefit future research on tDCS.

Gait and balance deficits are a hallmark of disease progression [69]. These deficits eventually become refractory motor complications and can lead to disability among patients with PD [45]. In advanced stages of PD, patients typically exhibit abnormal gait patterns such as reduced gait speed and step length, increased cadence, and double-limb support [70, 71]. Posture control when standing up, the narrowing of the support base while walking, and postural instability in the mediolateral plane when turning worsen as PD progresses [72]. Additionally, FOG commonly occurs when patients walk, turn, and traverse narrow hallways, all of which increase fall risk [71, 72]. These gait and balance impairments arise from various pathological mechanisms involving the BG network [73]. As a clinically noninvasive brain stimulation procedure, tDCS effectively rehabilitates gait and balance and produces noticeable results by applying an anodal charge over the targeted cortex. The beneficial effects of tDCS on gait and balance can be explained by two mechanisms. Applied anodal tDCS on motor cortices could induce dopamine releases in the BG by activating glutamatergic corticostriatal projections and could modulate the functional connectivity in corticostriatal and thalamocortical circuits. Most studies took advantage of the immediate mechanisms of tDCS and supplied a single session of tDCS to examine short-term improvements. However, it should be noted that the positive changes in gait and balance after tDCS were inconsistent with the stimulation area and intensity. In one study by Wong et al. [44], tDCS was applied separately over the primary motor cortex (M1), dorsolateral prefrontal cortex (DLPFC), and the cerebellum within 20 min. Despite the differences between pre- and post-intervention found in the majority of gait parameters (gait speed, cadence, and step length), none of the groups exhibited significant differences, including the sham group. Their results also supported that tDCS targeting M1 or DLPFC can improve gait in a single walking task. Another study compared single-target (M1) and multitarget (M1 and DLPFC) tDCS protocols. This study indicated that simultaneously stimulating M1 and DLPFC at an intensity of 1.5 mA for 20 min, rather than only M1, was more effective in alleviating FOG severity and balance, which was reflected by gait speed and TUG test results [43]. Another study performed anodal tDCS over M1 with 1 mA, 2 mA, and sham tDCS during separate 20-min sessions [46]. A better postural response to external perturbations among patients with PD was observed for 2 mA but not for 1 mA or sham. These observations demonstrated the substantial heterogeneity in tDCS protocols employed across the included study. Accordingly, it is critically important to establish investigations that focus on optimizing tDCS treatment protocols and investigating whether these various parameters have a notable influence on the effects of tDCS.

Regarding the combination of tDCS and other therapies, the action mechanism of tDCS could promote the inherent positive effects of rehabilitation therapies on motor performances in patients with PD. Kaski et al. [41] revealed that applying both tDCS and physical training was more effective in improving gait functions than training or tDCS alone. Furthermore, Conceicao et al. determined that the gait variability, executive control of walking and processing speed were enhanced by applying one session of anodal tDCS during aerobic exercise [63]. This study also highlighted that the addition of tDCS to aerobic exercise could modulate cholinergic activity, which affects gait disturbances in patients with PD. Additionally, numerous studies in our meta-analysis have confirmed that combined gait and balance training with tDCS improved gait speed [41, 59], stride length [41], double support time [59], cadence and step length [55, 60], TUG test results [56, 59], and BBS scores [55]. These findings support the benificial effects of tDCS with other therapies on gait and balance among patients with PD.

There are a number of limitations listed in the current study. First, half of the included studies were crossover designs with a 1-week washout that may have resulted in a carry-over effect. Nevertheless, the effect of tDCS would not be prolonged for a substantial period. Second, the validity of our results may be influenced by the fact that most of the included studies had a small number of participants. Third, many studies did not report using an intention-to-treat analysis or having allocation concealment or blinding (including participant, therapist, and assessor), which could have produced biases in the original studies and influenced the results of this meta-analysis. Fourth, the variety of tDCS protocols, such as intervention length, electrode montages, and additional therapies, may have affected the consistency among studies. Fortunately, no significant heterogeneity was observed in any analysis. Fifth, we were unable to investigate the effects of tDCS on each stage of the disease due to substantial variation in disease severity and the insufficient data reported in the included studies. Finally, the effects of tDCS on gait and balance were moderate, but effect sizes were almost entirely smaller than 0.5 and, in some cases, did not significantly differ from the control group. Therefore, future studies could further investigate under a larger sample size and be more methodologically rigorous when studying the effects tDCS in individuals with PD.

## Conclusions

Gait and balance impairments are incredibly challenging to address in PD rehabilitation. tDCS is an adjuvant treatment that has demonstrated benefits for improving motor and non-motor functions in PD patients. The results of our systematic review and meta-analysis offer substantial evidence that tDCS, whether used alone or in combination with other therapies, significantly enhances gait and balance in individuals with PD compared to sham tDCS or sham tDCS combined with other therapies. Nevertheless, it is crucial to recognize that the optimal protocol for tDCS in the treatment of PD has not yet been established. Consequently, further research is essential to identify the therapeutic protocols that are critical for maximizing the efficacy of tDCS.

### Clinical implication

To date, growing evidence uncovers the potential benefits of tDCS in various neurological conditions, including PD. It is thus becoming more critical to incorporate its significance into therapeutic practice. Our systematic review and meta-analysis of tDCS effects are practically meaningful to research and clinical applications. Our study conclusively demonstrates that tDCS, whether used alone or in combination with other therapies, is efficacious in improving certain aspects of gait and balance in individuals with PD. These findings hold significant clinical relevance as they inform healthcare decision-making for clinicians and patients, shedding light on the advantages and therapeutic benefits of tDCS among a variety of existing non-invasive brain stimulation techniques. In particular, these findings facilitate the integration of tDCS as a valuable component within a comprehensive PD rehabilitation program. However, it is essential to note that the optimal protocol of tDCS is not yet established for treating PD. Therefore, further research is necessary to elucidate the specific protocol, including targeted area, intensity, duration, and targeted stage of the disease, to maximize the benefit impacts of tDCS.

### Supplementary Information

**Additional file 1: Table S1.** PRISMA Checklist 2020.

**Additional file 2: Figure S1.** Forest plot of standardized mean difference (SMD) and their 95% CI for step length. **Figure S2.** Forest plot of standardized mean difference (SMD) and their 95% CI for walking time. **Figure S3**. Forest plot of standardized mean difference (SMD) and their 95% CI for stride time. **Figure S4.** Forest plot of standardized mean difference (SMD) and their 95% CI for double support time.

**Additional file 3: Figure S5.** Funnel plot of gait speed. **Figure S6.** Funnel plot of stride length. **Figure S7.** Funnel plot of cadence.

**Additional file 4: Figure S8.** Forest plot of standardized mean difference (SMD) and their 95% CI for dynamic gait index.

**Additional file 5: Figure S9.** Funnel plot of timed up and go test.

## Data Availability

All data generated or analyzed during this study are included in this published article and its Additional information files.

## Abbreviations

PD: Parkinson’s disease
BG: Basal ganglia
BG–Ctx–Cer: Basal, cortex, and cerebellum
M1: Primary motor cortex
DLPFC: Dorsolateral prefrontal cortex
tDCS: Transcranial direct current stimulation
tES: Transcranial electrical stimulation
rTMS: Repetitive transcranial magnetic stimulation
atDCS: Anodal tDCS
FOG-Q: Freezing of gait questionnaire
10MWT: Ten-meter walking test
6MWT: Six minutes waking test
CoP: Center of pressure
TUG: Timed up and go test
BBS: Berg balance scale
BESTest: Balance evaluation systems test
FRT: Functional reach test
DGI: Dynamic gait index
FGA: Functional gait assessment
UPDRS III: Unified Parkinson Disease Rating Scale Motor section
H&Y score: Hoehn & Yahr score
RCT: Randomized control trial
SMD: Standardized mean difference
CI: Confidence interval
SD: Standard deviation
IG: Intervention group
CG: Control group
