# Supplementary material for: Effects of transcranial direct current stimulation alone and in combination with rehabilitation therapies on gait and balance among individuals with Parkinson’s disease: a systematic review and meta-analysis
Source: J Neuroeng Rehabil. 2024 Feb 19;21:27. doi: 10.1186/s12984-024-01311-2 (PMC10875882; doi:10.1186/s12984-024-01311-2)
Supplement: Supplementary file 2 — Additional file 2: Figure S1. Forest plot of standardized mean difference (SMD) and their 95% CI for step length. Figure S2. Forest plot of standardized mean difference (SMD) and their 95% CI for walking time. Figure S3. Forest plot of standardized mean difference (SMD) and their 95% CI for stride time. Figure S4. Forest plot of standardized mean difference (SMD) and their 95% CI for double support time. [file 12984_2024_1311_MOESM2_ESM.docx]

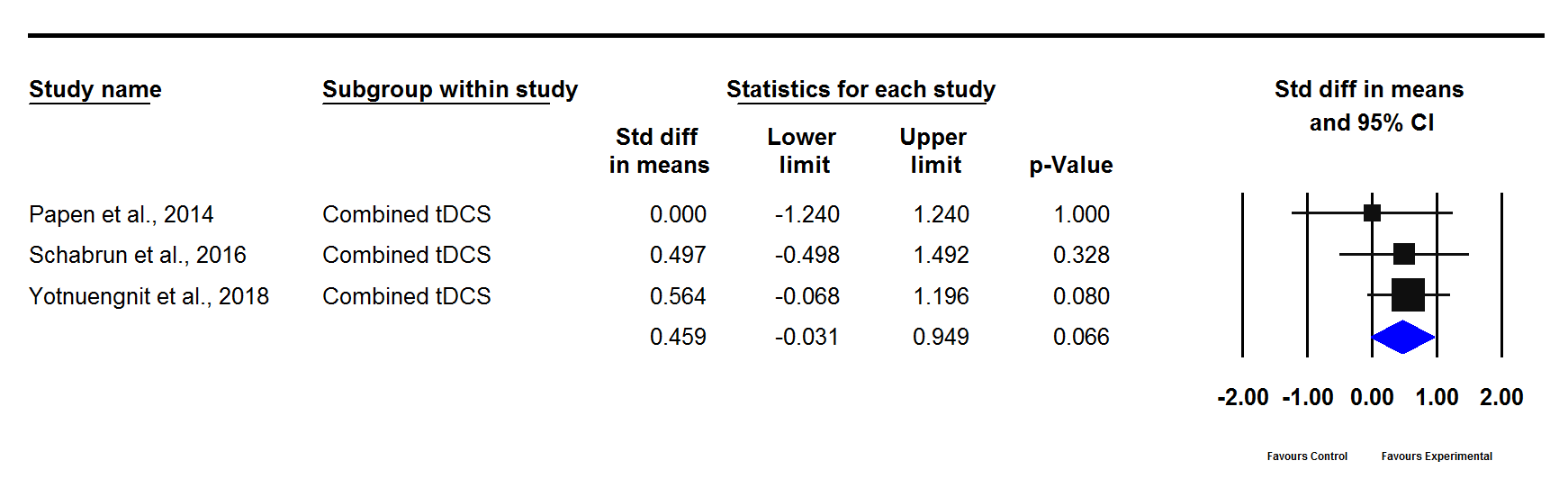


**Figure S1. Forest plot of standardized mean difference (SMD) and their 95% CI for step length.**

Note: Black squares represent the SMD in individual trials. The bottom blue rhombus indicates an overall pooled effect. Horizontal lines represent 95% confidence interval (CI). tDCS: Transcranial direct current. Although patients who underwent tDCS and rehabilitation therapies increased step length during walking, no significant differences were found compared with patients who only underwent rehabilitation therapies (*P* = 0.066).


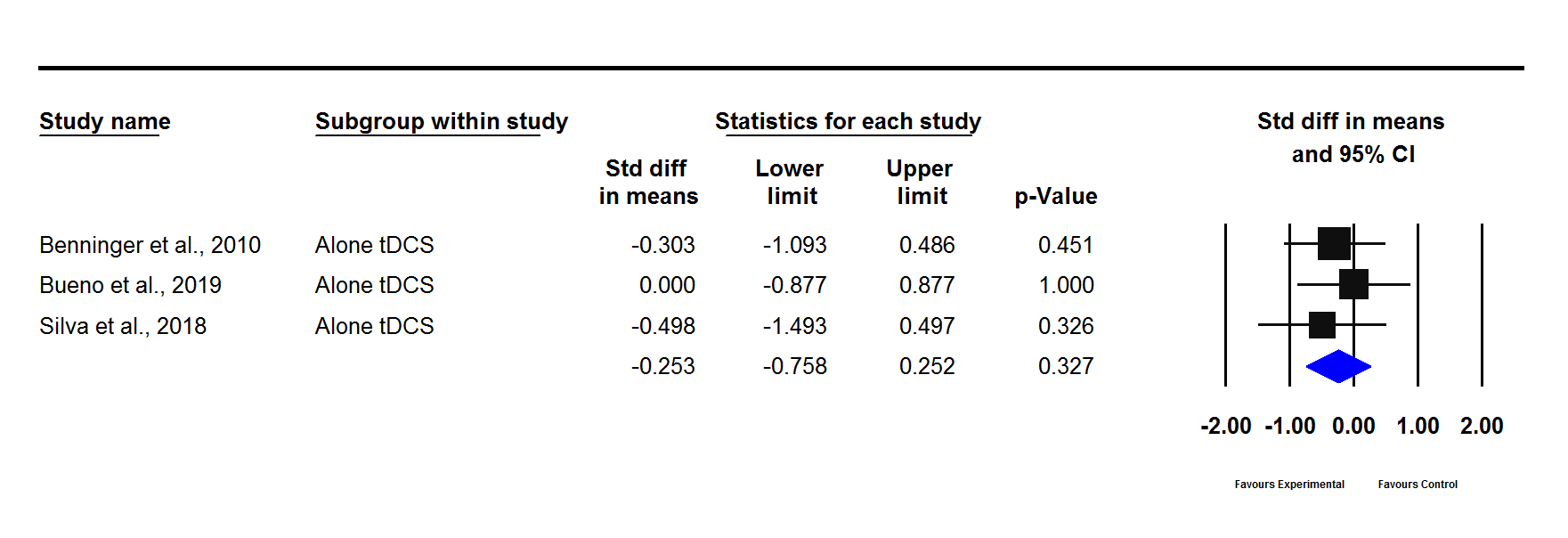


**Figure S2. Forest plot of standardized mean difference (SMD) and their 95% CI for walking time.**

Black squares represent the SMD in individual trials. The bottom blue rhombus indicates an overall pooled effect. Horizontal lines represent 95% confidence interval (CI). tDCS: Transcranial direct current stimulation. Real tDCS can reduce walking in PD patients, but this reduction is not a statistically different from sham tDCS (*P* = 0.327).


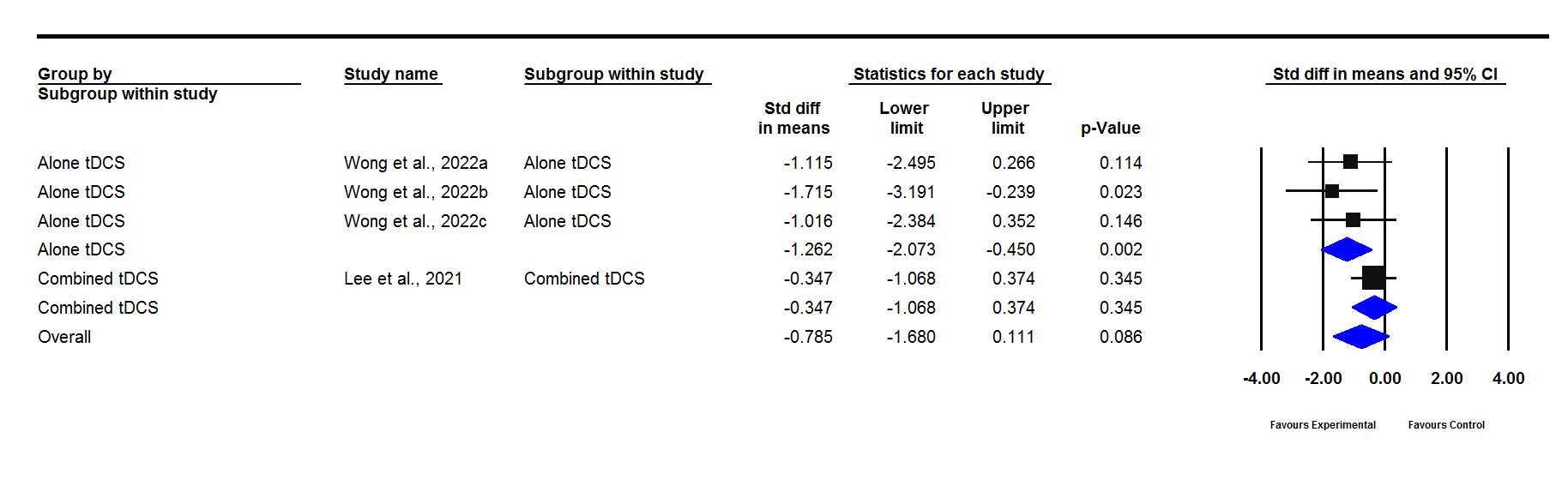


**Figure S3. Forest plot of standardized mean difference (SMD) and their 95% CI for stride time.**

Black squares represent the SMD in individual trials. The bottom blue rhombus indicates an overall pooled effect. Horizontal lines represent 95% confidence interval (CI). tDCS: Transcranial direct current stimulation. Compared with sham tDCS, real tDCS remarkable reduce stride time during walking of PD patients (*P* = 0.002). However, patients who received real tDCS plus additional therapies did not report a significant change in stride time compared to patients who received sham tDCS combined with additional therapies (*P* = 0.345).


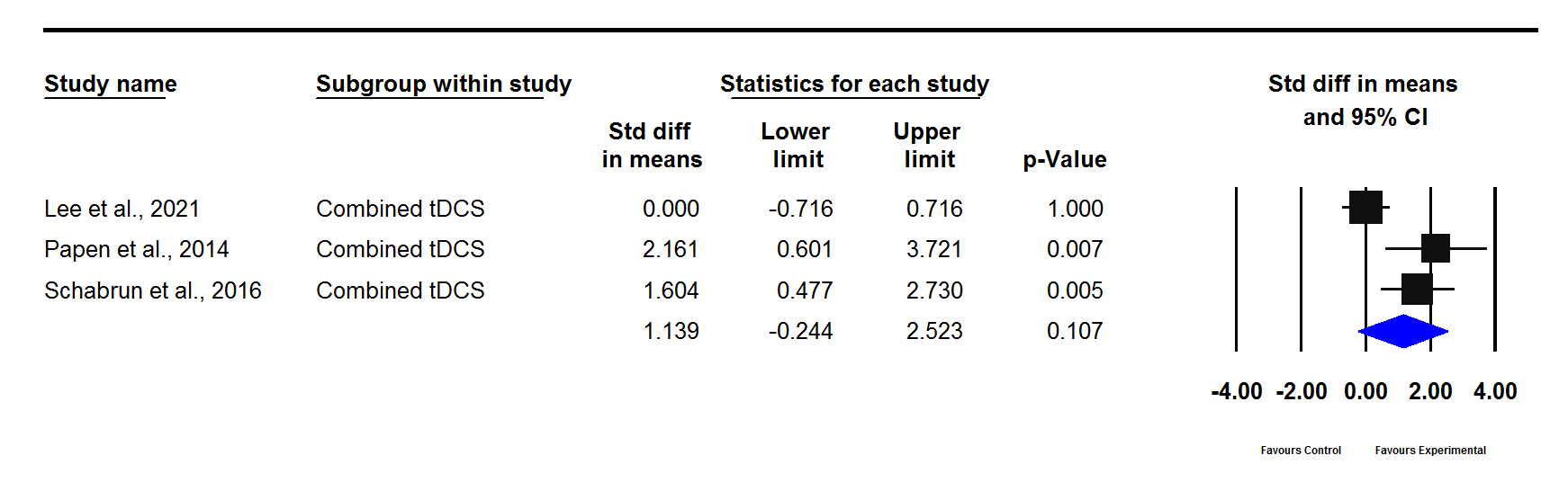
 **Figure S4. Forest plot of standardized mean difference (SMD) and their 95% CI for double support time.**

Black squares represent the SMD in individual trials. The bottom blue rhombus indicates an overall pooled effect. Horizontal lines represent 95% confidence interval (CI). tDCS: Transcranial direct current stimulation. Although patients who underwent tDCS and rehabilitation therapies reduced double support time in gait, no significant differences were found compared with patients who only underwent rehabilitation therapies (*P* = 0.107).
