# Supplementary material for: Effects of transcranial direct current stimulation alone and in combination with rehabilitation therapies on gait and balance among individuals with Parkinson’s disease: a systematic review and meta-analysis
Source: J Neuroeng Rehabil. 2024 Feb 19;21:27. doi: 10.1186/s12984-024-01311-2 (PMC10875882; doi:10.1186/s12984-024-01311-2)
Supplement: Supplementary file 3 — Additional file 3: Figure S5. Funnel plot of gait speed. Figure S6. Funnel plot of stride length. Figure S7. Funnel plot of cadence. [file 12984_2024_1311_MOESM3_ESM.docx]

**Figure S5. Funnel plot of gait speed.**


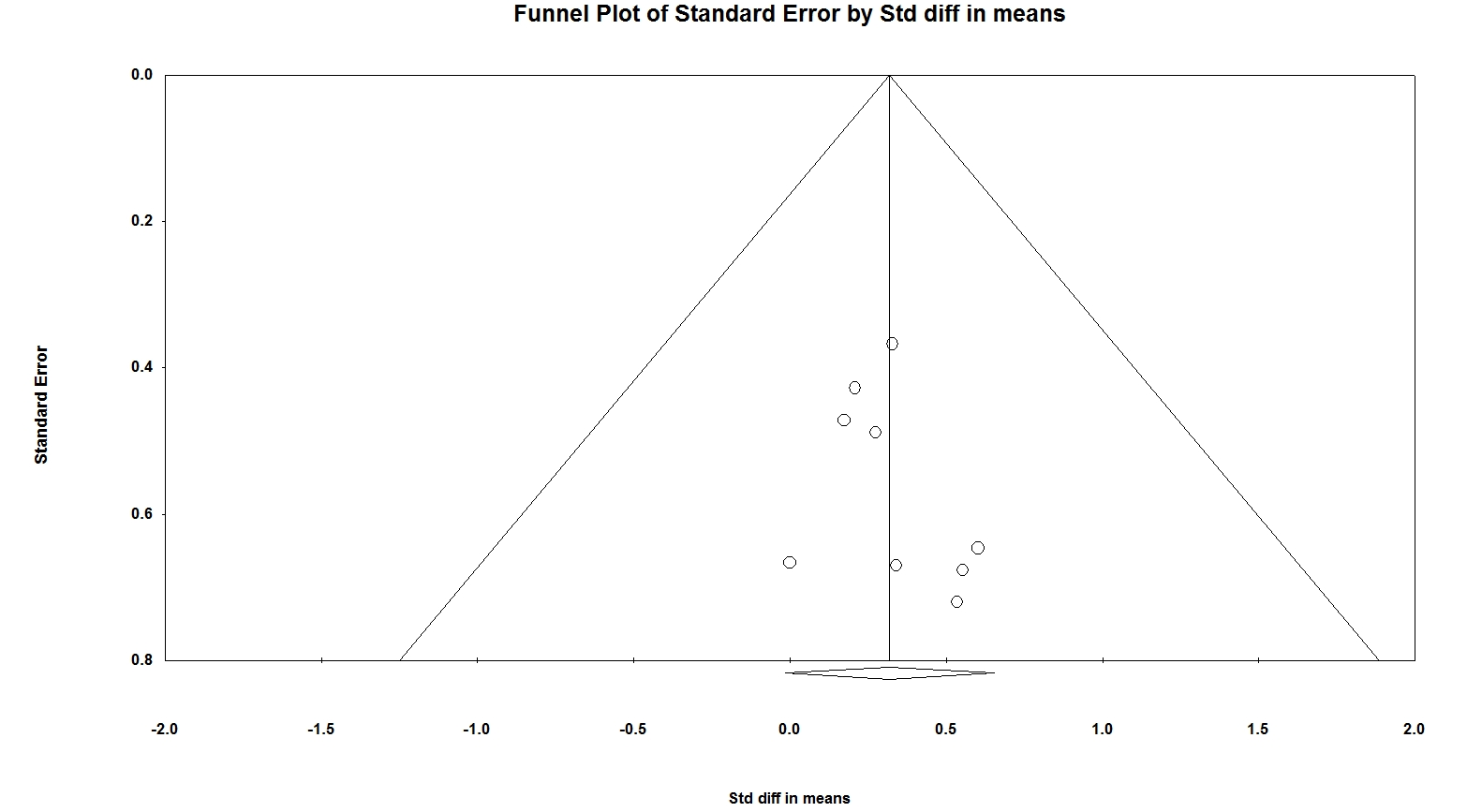


**Figure S6. Funnel plot of stride length.**


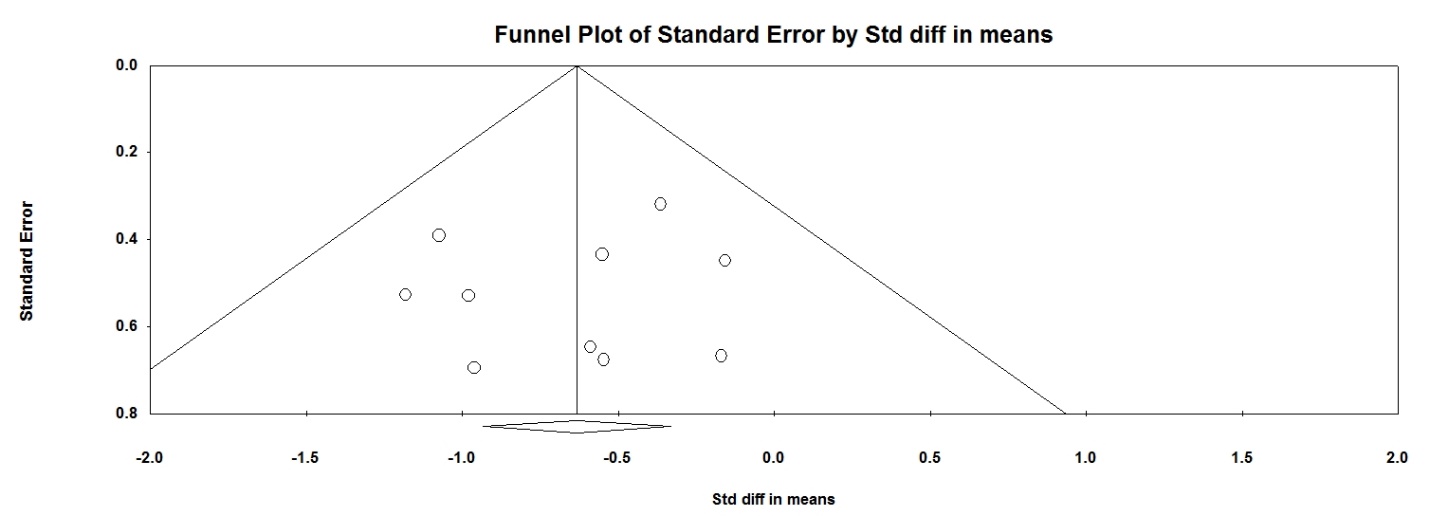


**Figure S7. Funnel plot of cadence.**
