# Supplementary material for: Effects of transcranial direct current stimulation alone and in combination with rehabilitation therapies on gait and balance among individuals with Parkinson’s disease: a systematic review and meta-analysis
Source: J Neuroeng Rehabil. 2024 Feb 19;21:27. doi: 10.1186/s12984-024-01311-2 (PMC10875882; doi:10.1186/s12984-024-01311-2)
Supplement: Supplementary file 4 — Additional file 4: Figure S8. Forest plot of standardized mean difference (SMD) and their 95% CI for dynamic gait index. [file 12984_2024_1311_MOESM4_ESM.docx]

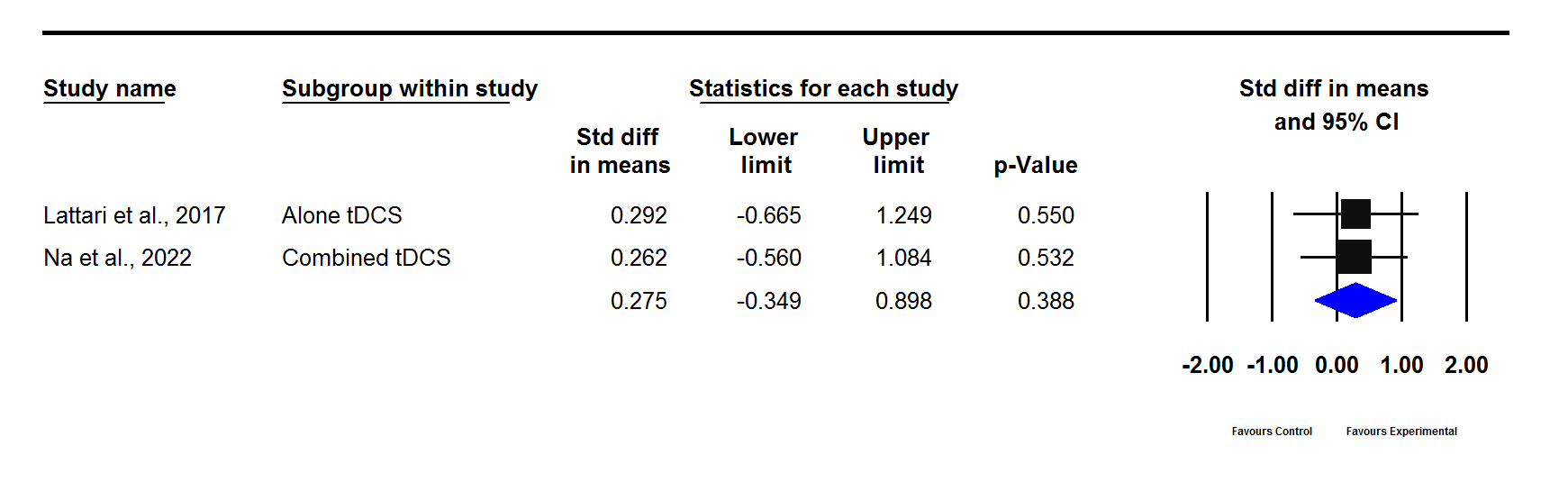


**Figure S8. Forest plot of standardized mean difference (SMD) and their 95% CI for dynamic gait index.**

Black squares represent the SMD in individual trials. The bottom blue rhombus indicates an overall pooled effect. Horizontal lines represent 95% confidence interval (CI). tDCS: Transcranial direct current stimulation. Compared with patients in the sham group, patients who received either tDCS alone or tDCS combined with additional rehabilitation therapies did not show a significant change in dynamic gait index (*P* = 0.388).
