# Supplementary figures and images for: Effects of transcranial direct current stimulation alone and in combination with rehabilitation therapies on gait and balance among individuals with Parkinson’s disease: a systematic review and meta-analysis
Source: J Neuroeng Rehabil. 2024 Feb 19;21:27. doi: 10.1186/s12984-024-01311-2 (PMC10875882; doi:10.1186/s12984-024-01311-2)

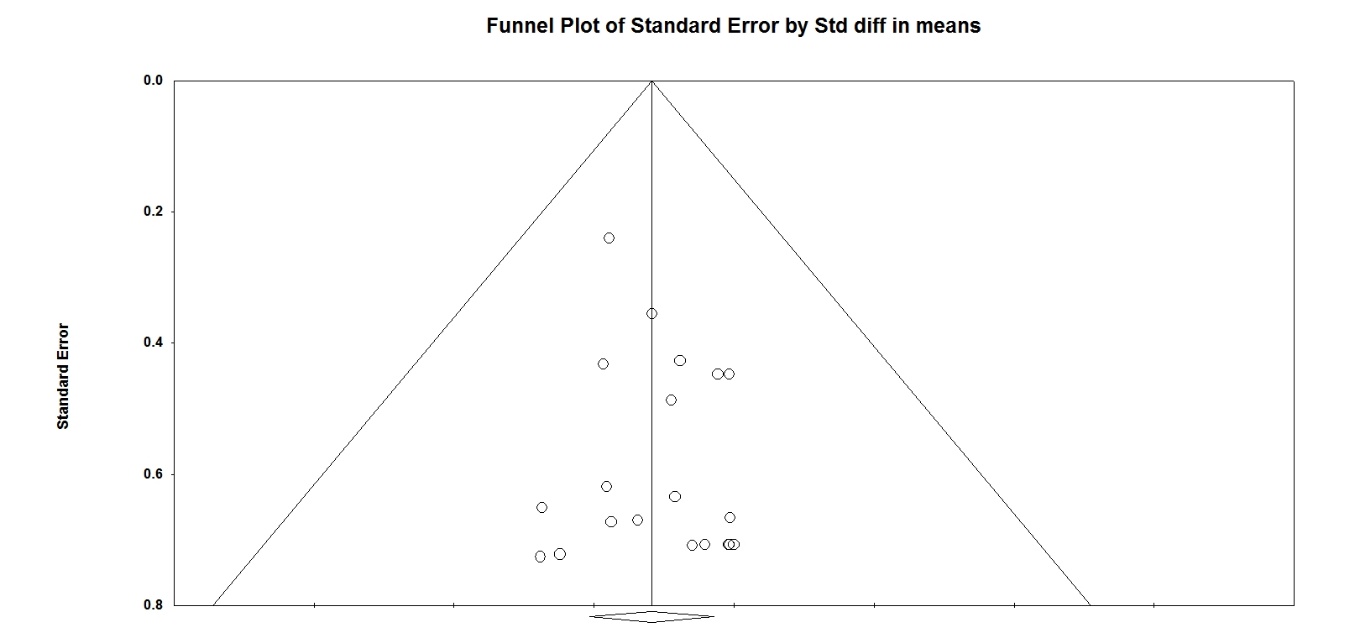


**Figure S9. Funnel plot of timed and go test.**

Supplement: Supplementary file 5 — Additional file 5: Figure S9. Funnel plot of timed up and go test. [file 12984_2024_1311_MOESM5_ESM.docx]
